# Metabolic Tumor Microenvironment Characterization of Contrast Enhancing Brain Tumors Using Physiologic MRI

**DOI:** 10.3390/metabo11100668

**Published:** 2021-09-29

**Authors:** Andreas Stadlbauer, Franz Marhold, Stefan Oberndorfer, Gertraud Heinz, Max Zimmermann, Michael Buchfelder, Elisabeth Heynold, Thomas M. Kinfe

**Affiliations:** 1 Institute of Medical Radiology, University Clinic St. Pölten, Karl Landsteiner University of Health Sciences, 3100 St. Pölten, Austria; Gertraud.Heinz@stpoelten.lknoe.at; 2 Department of Neurosurgery, Friedrich-Alexander University Erlangen-Nürnberg, 91054 Erlangen, Germany; Max.Zimmermann@med.uni-tuebingen.de (M.Z.); Michael.Buchfelder@uk-erlangen.de (M.B.); Elisabeth.Heynold@uk-erlangen.de (E.H.); thomasmehari.kinfe@uk-erlangen.de (T.M.K.); 3 Department of Neurosurgery, University Clinic of St. Pölten, Karl Landsteiner University of Health Sciences, 3100 St. Pölten, Austria; franz.marhold@stpoelten.lknoe.at; 4 Department of Neurology, University Clinic of St. Pölten, Karl Landsteiner University of Health Sciences, 3100 St. Pölten, Austria; Stefan.Oberndorfer@stpoelten.lknoe.at; 5 Department of Preclinical Imaging and Radiopharmacy, University of Tübingen, 72076 Tübingen, Germany; 6 Division of Functional Neurosurgery and Stereotaxy, Friedrich-Alexander University (FAU) Erlangen-Nürnberg, 91054 Erlangen, Germany

**Keywords:** brain tumors, hypoxia, neovascularization, physiological MRI, tumor microenvironment, energy metabolism, metabolic imaging

## Abstract

The tumor microenvironment is a critical regulator of cancer development and progression as well as treatment response and resistance in brain neoplasms. The available techniques for investigation, however, are not well suited for noninvasive in vivo characterization in humans. A total of 120 patients (59 females; 61 males) with newly diagnosed contrast-enhancing brain tumors (64 glioblastoma, 20 brain metastases, 15 primary central nervous system (CNS) lymphomas (PCNSLs), and 21 meningiomas) were examined with a previously established physiological MRI protocol including quantitative blood-oxygen-level-dependent imaging and vascular architecture mapping. Six MRI biomarker maps for oxygen metabolism and neovascularization were fused for classification of five different tumor microenvironments: glycolysis, oxidative phosphorylation (OxPhos), hypoxia with/without neovascularization, and necrosis. Glioblastoma showed the highest metabolic heterogeneity followed by brain metastasis with a glycolysis-to-OxPhos ratio of approximately 2:1 in both tumor entities. In addition, glioblastoma revealed a significant higher percentage of hypoxia (24%) compared to all three other brain tumor entities: brain metastasis (7%; *p* < 0.001), PCNSL (8%; *p* = 0.001), and meningioma (8%; *p* = 0.003). A more aggressive biological brain tumor behavior was associated with a higher percentage of hypoxia and necrosis and a lower percentage of remaining vital tumor tissue and aerobic glycolysis. The proportion of oxidative phosphorylation, however, was rather similar (17–26%) for all four brain tumor entities. Tumor microenvironment (TME) mapping provides insights into neurobiological differences of contrast-enhancing brain tumors and deserves further clinical cancer research attention. Although there is a long roadmap ahead, TME mapping may become useful in order to develop new diagnostic and therapeutic approaches.

## 1. Introduction

Brain tumors represent solid neoplasm inside the skull that may have developed from the brain tissue itself (primary brain tumors), other tissues such as the lymphatic tissue (primary central nervous system lymphomas, PCNSLs) or from the membranes that envelop the brain, the meninges (meningiomas). In addition, brain tumors may arise as a result of the spread of cancers primarily located in other parts of the body, which are also known as secondary brain tumors or brain metastases [1]. Among the heterogenic population of brain tumors, glioblastoma (GBM) remains the most common and most aggressive primary brain tumor in adults. GBM, PCNSL, brain metastases, and meningiomas together represent a large proportion of brain tumors encountered in clinical neurooncology. For instance, GBMs comprise 40% to 50% of primary brain tumors in adults, while PCNSL comprise up to 4% of primary CNS tumors, with an additional small contribution of secondary CNSL [2,3]. Brain metastases are found in 10% to 30% of adult neurooncologic patients with cancer at another location in the body, and nearly half of cases became clinical apparent as solitary metastases on clinical imaging [4,5]. Meningiomas are among the most common intracranial tumors, with an estimated incidence of eight cases per 100,000 persons per year [6,7] with the common type (meningioma WHO grade I) as a slow developing benign tumor [8].

Pretreatment characterization and differentiation of brain tumors using magnetic resonance imaging (MRI) is still a challenging diagnostic issue in clinical neurooncological practice as they may show very similar appearance on conventional MRI data as solitary strongly contrast-enhancing brain tumors surrounded by an edema visible as hyperintense area on T2-weighted MRIs [9,10,11]. A precise and reliable initial diagnosis is essential in order to initiate subsequent appropriate treatment management that can differ substantially depending on the type of a lesion [12,13]. The current standard of treatment for newly diagnosed GBM constitutes of maximal possible resection of the tumor, followed by adjuvant radiotherapy and chemotherapy with temozolomide [14]. Despite tumor biopsy, PCNSLs should not undergo a total gross resection as these malignancies are highly responsive to steroids and high-dose methotrexate-based chemotherapy alone or in combination with whole-brain radiation therapy [15,16,17,18]. Stereotactic radiosurgery, however, is considered an effective strategy in the treatment of brain metastases with the advantage of excellent local control rates with minimal invasiveness [19]. In the case of meningiomas, surgical resection has been recommended in tumors already causing symptoms, permitting observation with close follow-up MRIs if the meningioma is small and asymptomatic [20]. Consequently, accurate preoperative differentiation of contrast-enhancing brain tumors is critical for the individualized therapeutic decision making.

Hence, understanding the pathophysiology of brain tumors including the substantial inter- and intratumoral heterogeneity is essential for the development of both new diagnostic and therapeutic approaches [21,22,23]. Accumulating evidence suggests that microanatomical compartments present specific niches within the tumor microenvironment (TME) that regulate metabolic pathways, immune surveillance, survival, and invasion [24]. The TME has been recognized as a pivotal regulator of cancer development and maintenance, progression, and therapeutic response in primary and metastatic brain neoplasms [25]. The landscape of TME is shaped by the intra-tumoral heterogeneity considering the extent and degree of necrosis, hypoxia and neovascularization in combination with the dominating metabolic pathway used by the tumor cells in order to promote proliferation and energy production [26]. In contrast to normal differentiated cells, which rely primarily on mitochondrial oxidative phosphorylation (OxPhos) to generate the energy needed for cellular processes, cancer cells may instead rely on aerobic glycolysis, a phenomenon termed “Warburg effect.” Aerobic glycolysis is a faster but inefficient way to generate adenosine 5′-triphosphate (ATP). However, the advantage how it confers to cancer cells in not fully understood but subject of intense research [27].

Given these variabilities, there is an urgent neurooncological need for noninvasive in vivo probing of the spatial heterogeneity and the temporal dynamics of TME compartments in order to enable a more precise diagnostic or conceptualize tailored therapies. The neurobiological interplay between oxygen metabolism, tissue hypoxia, tumor vascular architecture, and neovascularization activity is of crucial importance for tumor metabolism and biology. Most of the current available approaches, however, are not well suited for combined in vivo characterization in humans due to their invasiveness (electrodes), limited availability and high costs (^15^O_2_ positron emission tomography, PET), or low spatial resolution (near-infrared spectroscopy, NIRS). Glucose chemical exchange saturation transfer (CEST) MRI [28] and high-resolution MR spectroscopic imaging (MRSI) [29] partly overcome these disadvantages and provide additional insight into the energy metabolism of the tumor. A novel multiparametric MRI approach termed TME mapping has been introduced recently that may overcome these disadvantages and limitations, thus permitting for noninvasive classification of TME heterogeneity and dynamics [30]. Furthermore, TME mapping enables the assessment of the dominant metabolic traffic for energy production detection of tumor-supportive hypoxic and vascular niches in brain tumors [30]. In this study, we used this physiological MRI approach and hypothesized that TME mapping provides insight into pathophysiological differences of contrast-enhancing brain tumors of different origin.

## 2. Results

### 2.1. Patient Characteristics

The institutional brain MRI database searched for this study contained a total of 1400 MR examinations using the study protocol in 480 brain tumor patients. A total of 120 patients (59 females; 61 males; mean age 62.2 ± 12.0 years; 27–84 years) with newly diagnosed, untreated contrast-enhancing brain tumors satisfied the inclusion criteria: 64 patients (53%; 27 females; 37 males; mean age 62.8 ± 12.7 years; 31–84 years) had the diagnosis of a glioblastoma (World Health Organization [WHO] grade 4, isocitrate dehydrogenase [IDH] wild type) and 20 patients (17%; 11 females; 9 males; mean age 62.8 ± 12.7 years; 31–84 years) had a brain metastasis. In these 20 patients, the brain metastases originated in nine patients from lung cancer, in four patients from breast cancer, in two patients each from a melanoma or renal cancer, and in one patient each from a fibrosarcoma, bladder cancer, and colon cancer, respectively. Furthermore, 15 patients (13%; 7 females; 8 males; mean age 68.3 ± 8.1 years; 55–78 years) had a primary central nervous system lymphoma (PCNSL) and 21 patients (18%; 14 females; 7 males; mean age 57.3 ± 13.4 years; 27–82 years) had the diagnosis of a meningioma WHO grade I.

### 2.2. Physiological MRI and TME Mapping of Contrast-Enhancing Brain Tumors

Physiological MRI data acquisition and calculation of biomarker maps of oxygen metabolism (oxygen extraction fraction, OEF, and cerebral metabolic rate of oxygen, CMRO_2_) and tissue oxygen tension (PO_2_) as well as microvascular architecture (microvessel density, MVD, and vessel size index, VSI) and neovascularization activity (microvessel type indicator, MTI) was successfully performed for all 120 patients. For all cases, the data quality of the biomarker maps was sufficiently high enabling TME mapping.

**Figure 1:**
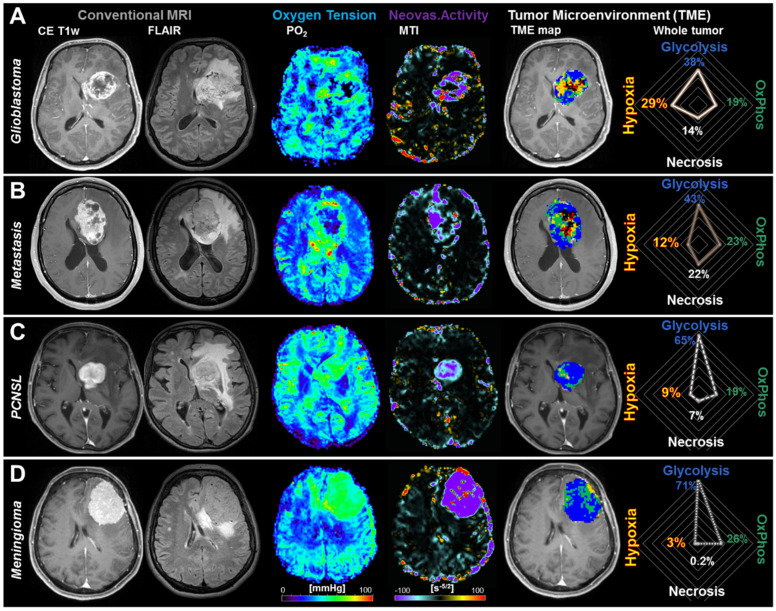
Tumor microenvironment (TME) mapping of patients with contrast-enhancing brain tumors. Conventional, anatomical MRI, tissue oxygen tension (PO_2_), neovascularization activity represented by MTI (microvessel type indicator), as well as the corresponding TME map and the distribution of the TME compartments for the whole tumor as radar chart for a patient with (**A**) glioblastoma WHO grade 4, IDH-wildtype; (**B**) a brain metastasis from breast cancer; (**C**) primary central nervous system (CNS) lymphoma (PCNSL); and (**D**) meningioma, respectively. Color code in the TME maps: blue = glycolysis, green = oxidative phosphorylation (OxPhos), black = necrosis, yellow and red = hypoxia with and without neovascularization, respectively. Note: hypoxia in the radar chart is the sum of hypoxia with neovascularization and hypoxia without neovascularization.

Figure 1A depicts an illustrative case for physiological MRI and TME mapping of a patient suffering from glioblastoma. In the PO_2_ map, the tumor showed very low oxygen tension in the central part of the tumor, which was surrounded by tumor parts with normal to high tissue oxygen tension. The MTI map showed a similar pattern, highly dysfunctional tumor neovascularization in the center surrounded by tumor parts with strong neovascularization activity. Interestingly, however, the areas were not completely congruent. Combination of this information and classification as TMEs revealed the full extent of metabolic intratumoral heterogeneity of the glioblastoma: a relatively small central necrosis (black area) was surrounded by extensive hypoxic areas without and with neovascularization (red and yellow areas). Vital tumor areas with aerobe glycolysis (blue) or oxidative phosphorylation (green) for energy production were mainly located at the border zone but also extended partly into the tumor center. The radar chart for the TME of the whole glioblastoma (at the far right) showed that two-thirds of the vital tumor were dominated by aerobic glycolysis (glycolysis:OxPhos = 38%:19%) and two-thirds of the rest of the tumor were dominated by hypoxia (total hypoxia:necrosis = 29%:14%). These findings underline the highly heterogeneous metabolic TME of glioblastoma.

A representative case for a patient suffering from a brain metastasis originating from breast cancer is illustrated in Figure 1B. Similar to glioblastoma, the tumor center showed low tissue oxygen tension and dysfunctional neovascularization surrounded by a tumor rim with increased oxygen tension and partly high neovascularization activity. The TME mapping, however, revealed differences between these two tumor entities: the tumor center is less hypoxic but more necrotic. The radar chart for the TME compartments of the whole metastasis showed that again approximately two-thirds of the vital tumor were dominated by aerobic glycolysis (glycolysis:OxPhos = 43%:23%), but approximately two-thirds of the tumor center were dominated by necrosis (total hypoxia:necrosis = 12%:22%) indicating that brain metastases may have a very heterogeneous metabolic TME, but are less hypoxic compared to glioblastomas.

An example for a patient with a PCNSL is presented in Figure 1C. This tumor showed high oxygen tension as well as intact and functional tumor neovascularization over most of the tumor area. TME mapping revealed that the tumor was dominated by aerobic glycolysis combined with some areas with oxidative phosphorylation for energy production. This resulted in a glycolysis-to-OxPhos ratio of 65%:19% or approx. 3:1 for the vital part of the PCNSL, which was higher compared with both the glioblastoma and the brain metastasis. Interestingly, the percentage of OxPhos (19%) in the metabolism of the total tumor volume was, however, similar to these tumor entities. The percentages of hypoxic or necrotic tumor tissue of the PCNSL were clearly lower compared to glioblastoma and brain metastasis.

Figure 1D presents a patient with a meningioma that demonstrated high tissue oxygen tension and neovascularization across the whole tumor. Similar to the PCNSL, the TME mapping revealed a glycolysis-to-OxPhos ratio of 71%:26% or again approx. 3:1 for the vital tumor part. The percentage of OxPhos (26%) on the whole tumor metabolism, however, was the highest for all four entities. Only few hypoxic areas and hardly any necrotic areas were detectable.

### 2.3. Differences in the Tumor Microenvironment between Contrast-Enhancing Brain Tumors

**Figure 2:**
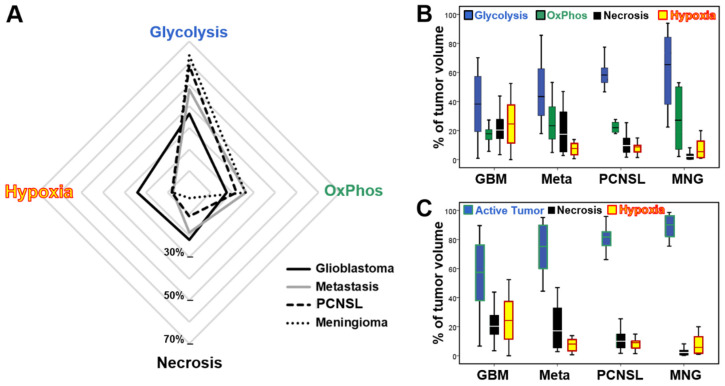
Comparison of the distribution of TME compartments in the different contrast-enhancing brain tumor entities. (**A**) Radar chart visualizing the distribution of the TME compartments with glycolysis, OxPhos, necrosis, and total hypoxia (sum of hypoxia with and without neovascularization) in the patient subgroups with glioblastoma (black line), brain metastasis (gray line), primary CNS lymphoma (PCNSL, dashed line), and meningioma (dotted line), respectively. (**B**) Box and whisker plots show the distribution of the TME compartments with glycolysis (blue), OxPhos (green), necrosis (black), and total hypoxia (yellow/red). (**C**) Box and whisker plots show the distribution of active tumor volume (i.e., the sum of glycolysis and OxPhos (blue/green), necrosis (black), and total hypoxia (yellow/red). Note: Boxes are mean ± SD, and whiskers are minimum and maximum values, respectively.

The radar chart for all 120 patients with newly diagnosed and untreated contrast-enhancing brain tumors is depicted in Figure 2A, which were largely in accordance with the results for the representative cases described above. Glioblastoma showed the highest intratumoral metabolic TME heterogeneity as well as the highest percentage of hypoxia (total hypoxia = 24%) and necrosis (22%). For brain metastasis, TME mapping also revealed a high metabolic heterogeneity with a similar percentage of necrosis but a clear lower percentage of hypoxic tumor tissue. Notably, for both tumor entities in vital tumor tissue the glycolysis-to-OxPhos ratio was approximately 2:1, i.e., two-thirds of the vital tumor used aerobic glycolysis for energy production.

Both PCNSL and meningioma showed a very high percentage of vital tumor tissue, i.e., low percentage of necrosis an especially hypoxia, with a higher glycolysis-to-OxPhos ratio of approximately 2.6:1 in vital tumor, i.e., more than 70% of the vital tumor used aerobic glycolysis for energy production. The percentage of oxidative phosphorylation, however, was rather similar for all four brain tumor entities. In other words the more aggressive the brain tumor, the higher the percentage of hypoxia and necrosis, and the lower the percentage of remaining vital tumor tissue and aerobic glycolysis were present.

This can be clearly seen from box-whisker plots in Figure 2B*,*C. Comparison of the percentages of active tumor as the sum of aerobic glycolysis plus oxidative phosphorylation (Figure 2C) revealed a clear trend of increasing percentage of active tumor and decreasing percentage of hypoxia and necrosis from glioblastoma to meningioma, i.e., correlating with the decreased aggressiveness of the brain tumor. Details of the percentages of the TME compartments for the four contrast-enhancing brain tumor entities are summarized in Table 1.

Statistical analyses using Welch’s analysis of variance (ANOVA) in combination with the Games-Howell post hoc test confirmed that the metabolically active tumor volume was significantly lower in glioblastoma compared to PCNSL (*p* = 0.001) and meningioma (*p* < 0.001). Furthermore, glioblastoma also showed a significant higher percentage of hypoxic tumor volume compared to all three other brain tumor entities: Brain metastasis (*p* < 0.001), PCNSL (*p* = 0.001), and meningioma (*p* = 0.003), respectively

## 3. Discussion

To improve and increase knowledge of the metabolic TME heterogeneity and dynamics of brain tumors is highly relevant for both, neurooncological clinical care and research. In this study, we used a multiparametric physiological MRI approach for noninvasive in vivo detection of metabolic TME compartments in order to assess differences in tumor neurobiology and to perform metabolic phenotyping of contrast-enhancing brain tumors. We demonstrated relevant distinct characteristics of the TME indicating indicative for diagnostic-supporting differences in the appearance of the four most common brain tumor entities that are inaccessible even with modern MRI methods currently available. We found that increasing aggressiveness of the brain tumor type, i.e., from meningioma to glioblastoma, was associated with an enhanced intratumoral heterogeneity of TME compartments due to higher degree of hypoxia and necrosis. However, this did not hold true for the percentage of OxPhos, which was found to be relatively constant among all four targeted brain tumor entities.

Hypoxic compartments in the TME are a known characteristic of many aggressive cancers including glioblastoma. High proliferation rates drive excessive oxygen demand, and tumor-related intravascular thrombosis and hemorrhage lead to dysfunctional neovascularization state, hypoperfusion, and eventually to nutrient deficiency and tissue hypoxia [31]. Furthermore, to sustain rapid proliferation, cancer cells metabolize glucose to lactate even in the presence of oxygen (i.e., aerobic glycolysis) for both energy production and generation of carbon molecules essential for cancer biosynthesis. This phenomenon, which is known as the Warburg effect [32], is in clear contrast to the energy metabolism of normal cells via oxidative phosphorylation (OxPhos) in mitochondria. Clinical observations indicate that the presence of necrosis in the TME may have a negative impact on prognosis and survival. For instance, Lacroix et al. [33] measured the extent of necrosis in glioblastoma on conventional anatomic MRIs and showed an inverse correlation of the degree of necrosis with patient survival.

To date, an increasing numbers of in-human studies have been published describing the application of multiparametric MRI in order to distinguish brain tumor heterogeneity. The vast majority of these articles investigated the usefulness of so-called advanced MRI methodologies in differentiating low-grade from high-grade gliomas or glioblastomas from brain metastases or meningiomas [34,35,36]. Several articles focused on differentiation between GBMs, metastases, and PCNSL [37,38,39,40,41,42] and achieved in part excellent diagnostic parameters for this purpose with an area under the receiver operating curve (AUC) of up to 0.98, sensitivity of 100%, specificity of 93%, and accuracy of 95% [43].

These studies mainly applied diffusion- and perfusion-weighted MRI in addition to conventional anatomical MRI in order to improve the diagnostic accuracy for brain tumor classification. The diffusion signal evaluated as apparent diffusion coefficient (ADC) values was used as a marker of cellular density [44] and the perfusion signal curves from conventional gradient echo dynamic susceptibility contrast (GE-DSC) evaluated as cerebral blood volume (CBV) values was used as marker for blood perfusion [45]. On the one hand, these measures give a very limited insight into the pathophysiology and TME of brain tumors, while on the other hand, the inclusion of ADC and CBV has been already standard in daily clinical routine diagnostics for several years and may no longer be referred to as “advanced MRI” but rather as conventional MRI. Furthermore, the focus of these MRI studies was solely on differentiating between brain tumor entities but not specifically targeting pathophysiological aspects or the metabolic heterogeneity of the TME. An increased knowledge of the metabolic compartments in the tumor microenvironment of brain tumors has so far mainly been derived from in vitro studies or from in vivo studies [46,47] using animal brain tumor models [48] that described adaptations of the energy metabolism with variable proportions of glycolysis and OxPhos in different TME compartments.

In this study, we used a combined physiological MRI approach to obtain information about oxygen metabolism and neovascularization to classify metabolic compartments in the TME relevant for brain tumor biology. In a previous study, TME mapping was applied in 52 patients with IDH wild type glioblastoma and uncovered two different metabolic phenotypes: A glycolytic dominant phenotype with stable functional tumor neovasculature, and a more necrotic/hypoxic dominant phenotype with high proportion of defective, dysfunctional tumor neovasculature. The latter phenotype displayed a more aggressive behavior, while patients with the glycolytic glioblastoma phenotype showed a longer progression free survival [30]. At recurrence after standard of care therapy, the glioblastoma demonstrated a switch from the initial metabolic phenotype, either from the glycolytic to the necrotic/hypoxic dominant phenotype or vice versa. Furthermore, a necrotic/hypoxic phenotype at recurrence was associated with a higher rate of multifocal distribution of the recurrent tumor [49]. TME mapping was used in a previous study [50] to monitor changes in the pathophysiology of recurrent glioblastoma in response to antiangiogenic treatment with bevacizumab and detected significant changes in the distribution of the TME compartments: All patients showed a decrease in active tumor volume and neovascularization as well as an increase in hypoxia and necrosis after 3 months. Furthermore, glioblastoma with a high percentage of neovascularization and active tumor before bevacizumab onset were associated with poor treatment responsiveness.

Several limitations in our study include the relatively small number of patients for brain tumor entity defined subgroups (20 brain metastases, 15 PCNSLs, 21 meningiomas). Therefore, study should be considered a comparative work between the individual tumor groups. We did not include glioblastoma with mutation of the IDH gene as well as astrocytoma or oligodendroglioma WHO grade 3 as additional subgroups. However, the IDH gene appears to be only mutated in about 10% of glioblastomas [51]. Astrocytoma and oligodendroglioma WHO grade 3, on the other hand, are tumor entities with different molecular genetic alterations (telomerase reverse transcriptase [TERT]) mutation and 1p19q co-deletion in oligodendroglioma) and prognosis, which in addition often show no significant contrast enhancement, which in turn have resulted in low scale cohorts in the respective subgroups. Hence, we decided exclude these patients from the study. Furthermore, we focused our analyses on the contrast enhancing tumor part and excluded the peritumoral brain zone. The highly aggressive nature of glioblastoma is also associated with their diffusely infiltration into the surrounding peritumoral regions that exceed the edges of the contrast enhancement. Brain metastases and meningioma, however, grow by displacement of the surrounding brain tissue, which is associated with purely vasogenic peritumoral edema [52]. Peritumoral edema could therefore also provide interesting insights into the pathophysiological differences between the brain tumors entities. The purpose of this study, however, was to investigate the metabolic TME compartments in the tumor core of brain neoplasms.

It is important to point out that the multiparametric quantitative blood-oxygen-level-depended (qBOLD) approach provides only an estimation of the oxygen metabolism with model-inherent limitations. The model assumes that the system is in the static dephasing regime [53] this leads to OEF values which are predominantly weighted to larger vessels averaged for the entire vasculature and ignores the intravascular component. Furthermore, accumulation of hemosiderin and/or proteins as well as other susceptibility effects (i.e., white matter fiber orientation or contrast agent leakage) and very low perfusion or avascularity (i.e., in necrosis; see also Equation (2) in the Methods section) could bias the OEF estimation [54,55,56]. Our TME mapping approach Our TME mapping approach also does not take into account glucose metabolism or lactate production. However, both are essential metabolites when assessing energy metabolism. In a further development of our TME mapping method, glucose CEST MRI [28] and high-resolution MR spectroscopic imaging [29] should provide the relevant information for a more complete mapping of the metabolic pathways.

Finally, we did not include a validation of our TME mapping approach. Biological validation of the MR-based parameters for PO_2_, CMRO_2_, hypoxia, and neovascularization, however, is required by correlation with findings from immunohistochemistry, invasive methods, or other metabolic imaging modalities such as PET [57,58] and metabolomics approaches (e.g., metabolic pathway assessment with ^13^C-labled glucose) [59]. These issues have to be addressed in future studies. Furthermore, individual tumor groups considering genetic and molecular subgroups (e.g., IDH and/or TERT mutation, 1p19q co-deletion, O^6^-methylguanine-DNA-methyltransferase [MGMT] status) should be investigated separately from each other in order to be able to go into more depth.

Conclusively, the fusion of physiological MRI biomarker information in combination with the classification of metabolic compartments of the TME enabled a deeper view into the pathophysiological differences between the brain tumor entities determined in our study. Our TME mapping method has a rather high spatial resolution, is non-invasive, and user-independent, which makes it a useful clinical research tool. Before this tool can be applied in clinical routine for differential diagnosis, treatment response assessment, or therapy monitoring, however, further studies are required that include a larger cohort and respective subgroups, histopathological correlations, the analysis of the peritumoral brain zone, and/or preclinical data for validation of the approach. In order to introduce the physiological MRI biomarkers and the TME approach into clinical routine diagnosis of brain tumor entities, methods of artificial intelligence (e.g., machine learning and convolutional neuronal networks) could helpful, which, however, requires further research.

## 4. Materials and Methods

### 4.1. Patients

The Ethics Committee of the Lower Austrian Provincial Government reviewed and approved the study protocol (protocol code GS1-EK-4/339-2015, date of approval: 29 February 2016). The study was conducted in accordance with the guidelines of the Declaration of Helsinki. Written informed consent was obtained from all included patients. A prospectively populated institutional brain MRI database was searched for patients with untreated contrast enhancing brain tumors that were newly diagnosed between February 2016 and April 2021. Further inclusion criteria were: (i) age ≥ 18 years; (ii) histopathological confirmation of one of the following brain tumor entities: glioblastoma (GBM, WHO grade 4) without mutation of the IDH gene (IDH wild type), brain metastasis, primary central nervous system lymphoma (PCNSL), or meningioma; (iii) no previous treatment of the brain tumor; (iv) MRI examinations with the study protocol.

### 4.2. MRI Data Acquisition

Acquisition of the MRI data with our study protocol was the first step of our TME mapping approach. All MRI examinations were performed on a clinical 3 Tesla scanner (Trio, Siemens, Erlangen, Germany) which was equipped with a standard 12-channel head coil. The conventional MRI protocol for clinical routine diagnosis of brain tumors included the following MRI sequences: (i) an axial fluid-attenuated inversion-recovery (FLAIR); (ii) an axial diffusion-weighted imaging (DWI) sequence; (iii) pre- and post-contrast enhanced (CE) high-resolution three-dimensional (3D) T1-weighted magnetization-prepared rapid acquisition with gradient echo (MPRAGE) sequences; and (iv) a gradient echo dynamic susceptibility contrast (GE-DSC) perfusion MRI sequence with 60 dynamic measurements during administration of 0.1 mmol/kg-bodyweight gadoterate-meglumine (Dotarem, Guerbet, Aulnay-Sous-Bois, France) at a rate of 4 mL/s using a MR-compatible injector (Medrad Spectris, Bayer HealthCare, Leverkusen, Germany). A 20-mL-bolus of saline was injected subsequently at the same rate. The parameters of the cMRI sequences are summarized in Table 2.

MRI data acquisition for investigation of tissue oxygen metabolism and tissue oxygen tension using the quantitative blood-oxygen-level-depended (qBOLD) imaging approach [60] included the following sequences: (i) a multi-echo gradient-echo sequence and (ii) a multi-echo spin-echo sequence for mapping of the transverse relaxation rates R_2_* (=1/T_2_*) and R_2_ (=1/T_2_), respectively.

For assessment of microvascular architecture and neovascularization activity with the vascular architecture mapping (VAM) approach [61], we additionally performed a spin-echo DSC (SE-DSC) perfusion MRI sequence conducted with the same parameters and contrast agent injection protocol as used for the routine GE-DSC perfusion MRI (Table 2). Our approach to minimize artefacts due to patient motion and differences in time to first-pass peak was described previously [62,63]. Furthermore, the SE-DSC perfusion MRI was performed prior to the GE-DSC perfusion MRI, which is beneficial in two ways because the SE echo-planar-imaging technique is less sensitive to contrast-agent leakage [64], and the first contrast agent injection for the SE-DSC perfusion MRI acts as a pre-bolus for leakage artefact reduction of the more leakage-sensitive GE-DSC perfusion MRI.

All qBOLD and VAM sequences were carried out with identical geometric parameters (voxel size, number of slices, etc.) and slice position as used for the routine GE-DSC perfusion sequence (Table 2). The additional data acquisition time for the qBOLD (R_2_* and R_2_-mapping: 1.5 and 3.5 min) and VAM sequences (SE-DSC perfusion: 2 min) was seven minutes.

**Figure 3:**
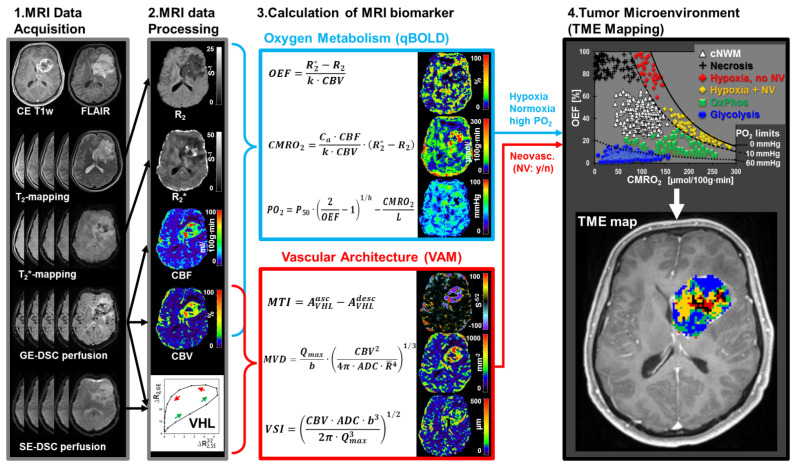
Work flow of the TME mapping method. Note: The TME map has the same color code as depicted in the OEF-CMRO_2_-scatterplot. The solid black line in this scatter plot indicates the isoline for PO_2_ = 0 mmHg, the dashed isoline for PO_2_ = 10 mmHg, and the dotted isoline for PO_2_ = 60 mmHg, respectively. These lines border the areas with hypoxia (PO_2_ between 0 and 10 mmHg), normoxia (PO_2_ between 10 and 60 mmHg), and high tissue oxygen tension (PO_2_ > 60 mmHg).

The subsequent steps of the TME mapping approach (2nd step: MRI data processing, 3rd step: calculation of MRI biomarkers, and 4th step: TME mapping) were performed with custom-made MatLab (MathWorks, Natick, MA, USA) software and are described in the following subchapters. The entire process is summarized in Figure 3.

### 4.3. MRI Data Processing

Processing of the conventional MRI data included calculation of the apparent diffusion coefficient (ADC) maps from DWI data using the following equation:

ADC = −ln[(S/S_0_)/b] (1)

Furthermore, absolute cerebral blood volume (CBV) and flow (CBF) maps from the GE-DSC perfusion MRI data were determined via automatic identification of arterial input functions (AIFs) [65,66].

MRI data processing for the qBOLD approach required corrections for background fields of the R_2_*-mapping data [67] and for stimulated echoes of the R_2_-mapping data [68] followed by calculation of R_2_*- and R_2_-maps from the multi-echo relaxometry data.

MRI data processing for the VAM technique included correction for remaining contrast agent extravasation as described previously [61,69,70]; fitting of the first bolus curves for each voxel of the GE- and SE-DSC perfusion MRI data with a previously described gamma-variate function [71], and calculation of the ∆R_2,GE_ versus (∆R_2,SE_)^3/2^ diagram [72], the so-called vascular hysteresis loop (VHL) [61,73].

### 4.4. Calculation of MRI Biomarkers

Calculation of MRI biomarker maps of oxygen metabolism including oxygen extraction fraction (OEF) and cerebral metabolic rate of oxygen (CMRO_2_) [60] as well as of tissue oxygen tension (PO_2_) [74,75] was performed using the following equations:

(2) $$\mathrm{OEF}=\frac{R_{2}^{*}{\;-\;R}_{2}}{\frac{4}{3}{\cdot \pi \cdot \gamma \cdot \Delta \chi \cdot \mathrm{Hct}\cdot B}_{0}\cdot \mathrm{CBV}}$$

with γ (2.67502 × 10^8^ rad/s/T) is the nuclear gyromagnetic ratio; Δχ = 0.264 × 10^−6^ is the difference between the magnetic susceptibilities of fully oxygenated and fully deoxygenated haemoglobin; Hct = 0.42 × 0.85 is the microvascular hematocrit fraction, whereby the factor 0.85 stands for a correction factor of systemic Hct for small vessels. The constant factors in the denominator were summarized as k in Figure 3;

(3) $${\mathrm{CMRO}}_{2}=\frac{C_{a}\;\cdot \;\mathrm{CBF}}{k\;\cdot \;\mathrm{CBV}}\;\cdot \;\left(R_{2}^{*}\;-\;R_{2}\right)$$

where C_a_ = 8.68 mmol/mL is the arterial blood oxygen content [76]; and

(4) $${\mathrm{PO}}_{2}{=p}_{50}\sqrt[{h}]{\left(\frac{2}{\mathrm{OEF}}-1\right)}-\frac{{\mathrm{CMRO}}_{2}}{L}$$

where p_50_ is the hemoglobin half-saturation tension of oxygen (27 mmHg), h is the Hill coefficient of oxygen binding to hemoglobin (2.7), and L (4.4 mmol/Hg per minute) is the tissue oxygen conductivity as defined by Vafaee and Gjedde [77].

For calculation of MRI biomarker maps of microvascular architecture [78] including microvessel density (MVD) and vessel size index (VSI, i.e., microvessel radius), the VHL curve data and the following equations were used:

(5) $$\mathrm{MVD}=\frac{Q_{\max}}{b}\cdot {\left(\frac{{\mathrm{CBV}}^{2}}{4\pi^{2}\cdot \mathrm{ADC}\cdot {\bar{R}}^{4}}\right)}^{1/3}$$

and

(6) $$\mathrm{VSI}={\left(\frac{\mathrm{CBV}\cdot \mathrm{ADC}\cdot b^{3}}{2\pi \cdot Q_{\max}^{3}}\right)}^{1/2}$$

with Q_max_ = max[∆R_2,GE_]/max[(∆R_2,GE_)^3/2^]; $\bar{R}$ ≈ 3.0 μm is the mean vessel lumen radius and b is a numerical constant (b = 1.6781) [78]. Finally, neovascularization activity estimated by the microvessel type indicator (MTI) was previously [61] defined as the area of the VHL curve signed with the rotational direction of the VHL curve, i.e., a clockwise VHL-direction was identified with a plus-sign, and a counter-clockwise VHL-direction was identified with a minus-sign [61]. Based on previous studies [61,79], a positive MTI value (i.e., a VHL curve in clockwise direction) is associated with a vascular system that is dominated by arterioles, whereas a negative MTI value (i.e., a VHL curve in counterclockwise direction) is associated with venule- and capillary-like vessel components. For guidance of interpretation of MTI maps, positive MTI values were assigned to warm colors and negative MTI values to cool colors. Therefore, MTI maps allow differentiation between supplying arterial (areas with warm colors) and draining capillary-venous (areas with cool colors) microvasculature, and high negative MTI values, are associated with strong neovascularization activity in the tumor [61].

### 4.5. Tumor Microenvironment Mapping

The approach for mapping of the tumor microenvironment [30] required quantitative classification of the MRI biomarker values for oxygen metabolism and microvascular architecture followed by fusion of this classified MRI biomarker information. This was performed in a voxel-by-voxel manner.

Firstly, each voxel of the imaging volume of interest was quantitatively classified regarding tissue oxygen tension status using the following PO_2_ limit values: hypoxia for PO_2_ < 10 mmHg; normal oxygen concentration for PO_2_ = 10–60 mmHg; and high oxygen concentration for PO_2_ > 60 mmHg. PO_2_ limit values for were obtained from contralateral normal appearing white matter (cNWM, white triangles in the CMRO_2_-OEF-scatterplot, downright) and from the literature [80,81,82]. 

Secondly, the tumor neovasculature was classified by using the following limit values: no neovascularization activity and/or dysfunctional tumor vasculature for MTI between ±5.0 s-5/2 and/or MVD < 250 mm-2; all other MTI and MVD values were classified as neovascularization activity and functional tumor vasculature. Limit values for MTI and MVD were again obtained from cNWM (white triangles) and from results of receiver-operating curve (ROC) analyses of a previous study [62].

Finally, the classified MRI biomarker information about the status of both oxygen metabolism and neovascularization was combined for each tumor voxel and classified as one of five tumor microenvironments. This classification was performed under consideration of the OEF-CMRO_2_-scatterplot (diagram at upright in Figure 3), which demonstrates that each TME has specific OEF-CMRO_2_-value pairs differing from cNWM (white triangles). These OEF-CMRO2-scatterplots were of central importance for classification of the five TME compartments, which were defined as follows (the assigned colors for the TME are given in brackets) [30]:

- Hypoxia without neovascularization or with dysfunctional tumor vasculature for voxels with high OEF, normal CMRO_2_ (associated with a low PO_2_ accordingly to Equation (4)), and low MTI: Red diamonds in the OEF-CMRO_2_-scatterplot and red voxels in the TME map (right-hand side in Figure 3).
- Hypoxia combined with neovascularization activity for voxels with normal to low OEF, high CMRO_2_ (associated with a low PO_2_), and high MTI: yellow diamonds in the OEF-CMRO_2_-scatterplot and yellow voxels in the TME map (right-hand side in Figure 3).
- Necrosis for voxels with very low CMRO_2_ and high OEF combined with highly defective tumor vasculature: Black crosses in the OEF-CMRO_2_-scatterplot and black voxels in the TME map (right-hand side in Figure 3).
- OxPhos for voxels with normal to low OEF, high CMRO_2_ (associated with normal PO_2_), and functional tumor neovasculature, under the assumption of predominantly mitochondrial oxidative phosphorylation for energy production: Green squares in the OEF-CMRO_2_-scatterplot and green voxels in the TME map (right-hand side in Figure 3).
- Glycolysis for voxels with low OEF, low CMRO_2_, (associated with high PO_2_), and functional tumor neovasculature, under the assumption of predominantly cytosolic aerobe glycolysis by the Warburg effect for energy production: Blue circles in the OEF-CMRO_2_-scatterplot and blue voxels in the TME map (right-hand side in Figure 3).

Each voxel of the imaging volume of interest was assigned with the respective color accordingly to the classification which resulted in the TME map (downright image in Figure 3). The limit values of the biomarker values for oxygen metabolism (CMRO_2_ and OEF, neovascularization activity (MTI), and microvascular architecture (MVD) for classification of the TME compartments are summarized in Table 3 for a better overview.

### 4.6. Quantitative and Statistical Analysis

For quantitative analysis, regions of interest (ROI) were manually defined based on features seen in the contrast-enhanced T1-weighted MRIs for the enhancing tumor region. The ROIs covered the whole enhancing tumor volume and were transferred to the TME maps. The volumes of the five TMEs as percentage of the whole tumor volume were calculated. Additionally, the active tumor volume as the sum of OxPhos and glycolysis was calculated.

Statistical analyses were performed using SPSS (version 21, IBM, Chicago, IL, USA) and R (version 3.6.3, R Foundation, Vienna, Austria). Significance of differences in TME volumes between the four different brain tumor entities was determined using the one-way analysis of variance (ANOVA) method. The Tukey test was used as post hoc procedure to be consistent with the assumption that homogeneity of variance was met and for correction for multiple comparisons. Homogeneity of variance was verified using the Levene’s test. When the assumption of homogeneity of variances was violated, Welch’s ANOVA in combination with the Games-Howell post hoc test was used. *p* values less than 0.05 were considered to indicate significance.

## Figures and Tables

**Table 1:** Distribution of the TME compartments for the four contrast-enhancing brain tumor entities.

	Glioblastoma	Metastasis	PCNSL	Meningioma
**Aerobic Glycolysis**	37% ± 22% 1–70%	48% ± 25% 18–86%	59% ± 10% 47–77%	63% ± 27% 22–94%
**Oxidative Phosphorylation**	17% ± 6% 1–27%	26% ± 17% 5–53%	22% ± 4% 18–28%	26% ± 22% 2–53%
**Vital Tumor**	54% ± 24% 7–90%	74% ± 18% 44–95%	81% ± 10% 66–96%	90% ± 9% 75–99%
**Necrosis**	22% ± 11% 3–44%	19% ± 17% 3–47%	11% ± 8% 2–25%	3% ± 3% 0–8%
**Hypoxia with Neovascularization**	15% ± 10% 0–36%	5% ± 4% 0–14%	5% ± 4% 0–11%	7% ± 7% 1–18%
**Hypoxia without Neovascularization**	9% ± 7% 0–27%	2% ± 2% 0–6%	3% ± 2% 0–6%	1% ± 1% 0–2%
**Total Hypoxia**	24% ± 16% 0–52%	7% ± 5% 1–14%	8% ± 5% 1–15%	8% ± 7% 1–20%

**Note**.—Vital Tumor is the sum of Aerobic Glycolysis plus Oxidative Phosphorylation; Total Hypoxia is the sum of Hypoxia with Neovascularization plus Hypoxia without Neovascularization. Primary central nervous system lymphoma PCNSL is primary CNS lymphoma.

**Table 2:** Sequence parameters of the MRI study protocol.

	Conventional MRI Sequences				Physiological MRI Sequences		
	FLAIR	MPRAGE	DWI	GE-DSC	SE-DSC	R_2_ * Mapping	R_2_ Mapping
**In-plane resolution**	0.45 × 0.45	1.0 × 1.0	1.2 × 1.2	1.8 × 1.8	1.8 × 1.8	1.8 × 1.8	1.8 × 1.8
**Slice thickness [mm]**	3.0	1.0	4.0	4.0	4.0	4.0	4.0
**Number of slices**	48	176	29	29	29	29	29
**TR [ms]**	5000	2100	5300	1740	1740	1210	3260
**TE [ms]**	460	2.3	98	22	33	5–40 ms	13–104 ms
Flip angle * [°]	120	12	90	90	90	90	90
**GRAPPA**	2	2	2	2	2	2	2
**other**	TI = 1800 ms		b = 0 and 1000 s/mm^2^	60 dynamic volumes	60 dynamic volumes	8 echoes	8 echoes

FLAIR, fluid-attenuated inversion-recovery; MPRAGE, magnetization-prepared rapid acquisition with gradient echo sequence for contrast-enhanced T_1_-weoghted MRI; DWI, diffusion-weighted imaging; GE-DSC, gradient echo dynamic susceptibility contrast perfusion MRI; SE-DSC, spin echo dynamic susceptibility contrast perfusion MRI; GRAPPA, parallel imaging using generalized autocalibrating partially parallel acquisition. * Flip angle means the angle of excitation. Refocusing angles were 180° for all sequences with a SE scheme, i.e., FLAIR, DWI, SE-DSC, and R_2_ mapping.

**Table 3:** Criteria for classification of TME compartments.

Description	Color Code	CMRO_2_ Range	OEF Range	MTI Limit	MVD Limit	PO_2_ Limit
	in TME Map	[µmol/100 g·min]	[%]	[s-5/2]	[mm-2]	[mmHg]
Hypoxia without NV	red	>80 and <150	>50	>−5.0 and <5.0	<250	<10
Hypoxia with NV	yellow	>150	<50	<−5.0 and >5.0	>250	<10
Necrosis	black	<130	>75	>−5.0 and <5.0	<250	n.a.
OxPhos with NV	green	>70	<50	<−5.0 and >5.0	>250	10−60
Glycolysis with NV	blue	<150	<20	<−5.0 and >5.0	>250	>60

NV, Neovascularization activity; n.a., not applicable, note: Very low perfusion or avascularity in necrosis could bias the PO_2_ estimation.

## Data Availability

Data available on request due to privacy and ethical restrictions.
